# The Down-Regulation of Circ_0059707 in Acute Myeloid Leukemia Promotes Cell Growth and Inhibits Apoptosis by Regulating miR-1287-5p

**DOI:** 10.3390/curroncol29090525

**Published:** 2022-09-18

**Authors:** Jichun Ma, Xiangmei Wen, Zijun Xu, Peihui Xia, Ye Jin, Jiang Lin, Jun Qian

**Affiliations:** 1 Department of Central Lab, Affiliated People’s Hospital of Jiangsu University, Zhenjiang 212050, China; 2 Zhenjiang Clinical Research Center of Hematology, Affiliated People’s Hospital of Jiangsu University, Zhenjiang 212050, China; 3 The Key Lab of Precision Diagnosis and Treatment in Hematologic Malignancies of Zhenjiang City, Affiliated People’s Hospital of Jiangsu University, Zhenjiang 212050, China; 4 Department of Hematology, Affiliated People’s Hospital of Jiangsu University, Zhenjiang 212050, China

**Keywords:** circular RNA, acute myeloid leukemia, circ_0059707, prognosis

## Abstract

Acute myeloid leukemia (AML) is the most common type of hematological malignancy. Recently, an increasing number of reports have shown that many circular RNAs can act as effective targets for AML. However, the roles of circ_0059707 in AML remain largely unclear. In this study, we found that the expression levels of circ_0059707 were significantly decreased in AML patients with respect to normal controls (*p* < 0.001). Low expression levels of circ_0059707 were also associated with a poor prognosis. Furthermore, circ_0059707 overexpression inhibited cell growth and promoted apoptosis in leukemia cells, compared with control cells. Circ_0059707- and empty plasmid-transfected cells were injected subcutaneously into BALB/c nude mice. We found that the tumor volume was significantly lower in mice in the circ_0059707 group than in control mice (*p* < 0.01). Nuclear pyknosis, nuclear fragmentation, nuclear dissolution, and cell necrosis were observed in the circ_0059707 group by HE staining. CircInteractome analysis showed that 25 microRNAs (miRNAs), including miR-1287-5p, ©-miR-1825, a©hsa-miR-326, may be potential targets for circ_0059707. The expression of these miRNAs was analyzed in both the GEO GSE51908 and the GSE142700 databases. miR-1287-5p expression was lower in AML patients compared with controls in both the GSE51908 and the GSE142700 datasets. Moreover, we demonstrated that miR-1287-5p expression was down-regulated in AML patients and up-regulated in circ_0059707-overexpressing cells. Collectively, our research demonstrated that the down-regulation of circ_0059707 was highly evident in de novo AML patients. Our analysis also demonstrated that circ_0059707 inhibited cell growth and promoted apoptosis by up-regulating miR-1287-5p.

## 1. Introduction

Acute myeloid leukemia (AML) is a form of malignant clonal disease of the hematopoietic system that originates in myeloid progenitor cells and is the most common type of acute leukemia [1,2]. Genetic changes, such as chromosomal abnormalities and gene mutations, play an important role in the development of AML [3,4,5]. Epigenetics changes are also known to be a pathological mechanism that plays an important role in the development of AML and include key genomic changes such as DNA methylation and non-coding RNA functions [6,7,8,9]. Although there are many therapeutic regimens for AML, the prognosis of patients affected by this disease is still unsatisfactory, and most patients will relapse and die after remission. Therefore, there is a clear and urgent need to identify new targets for the diagnosis, prediction, and treatment of AML, so to improve efficacy, monitoring, and treatment.

Epigenetic modifications associated with non-coding RNAs are known to be an important mechanism in the development of leukemia, and the discovery of this new form of RNA brings new hope to the treatment of many genetic conditions [7,10,11,12,13]. Circular RNA is a ubiquitous form of conservative non-coding RNA with characteristic stability [14]. Previous research reported that the knockdown of circMYBL2 inhibited the proliferation and promoted the differentiation of FLT3-ITD AML cells both in vivo and in vitro. The mechanism underlying these effects involves circMYBL2 increasing the binding of PTBP1 and FLT3 mRNA, improving the translation efficiency of the FLT3 kinase [15]. In a previous study, Wang et al. reported that circSPI1 acts as an oncogene by antagonizing SPI1 and by interacting with microRNAs in AML [16]. Other research showed that circRNF220 was associated with relapse, and the knockdown of circRNF220 inhibited cell proliferation and promoted apoptosis. This circular RNA may act as a sponge for miR-30a, thus affecting the levels of the miR-30a targets MYSM1 and IER2 [17].

circ_0059707 is a circular RNA formed by the cyclization of exon 2 in the process of ID1 cleavage. ID1 (inhibitor of DNA binding 1) is a negative regulator of HLH (helix–loop–helix) transcription factor that is widely expressed in the human body and acts as an oncogene to promote cell cycle progression, enhance proliferation and migration, and inhibit apoptosis [18,19,20,21]. Our group previously reported that ID1 was over-expressed in AML patients and that patients with high expression levels of ID1 had poor prognoses [22]. The overexpression of ID1 was also shown to promote the growth of leukemia cells, inhibit apoptosis, and increase resistance to decitabine. During splicing, ID1 forms two circular RNAs, circ_0059706 and circ_0059707, according to the circBase data base (http://www.circbase.org, 17 September 2022). In this study, we investigated the expression of circ_0059707 in AML patients, evaluated the clinical significance of circ_0059707 expression, and explored the role of circ_0059707 in the development of leukemia.

## 2. Materials and Methods

### 2.1. Patients

This study was approved by the Ethics Committee of the Affiliated People’s Hospital of Jiangsu University. A total of 117 participants, including 94 de novo AML patients and 23 controls, were enrolled in this study. All samples were obtained from the sample bank of our hospital, and all patients provided signed and informed consent. Bone marrow (BM) mononuclear cells were extracted from specimens of BM by gradient centrifugation (TBD Sciences, Tianjin, China). The diagnosis and classification of AML patients were based on French–American–British (FAB) and World Health Organization criteria [23,24]. Risk classification was based on European Leukemia Net guidelines [25].

### 2.2. BALB/c Nude Mice

Ten six-week-old specific pathogen-free (SPF) female BALB/c nude mice were purchased from Shanghai Slack Experimental Animal Co., Ltd. (license number: SCXK 2017-0005, Shanghai, China). All animal experiments were approved by the Experimental Animal Management and Use Committee of Jiangsu University. All mice were housed in SPF conditions, at room temperature (22 ± 2 °C) and humidity of 55 ± 5%. We injected 1 × 10^7^ cells subcutaneously into the back of each mouse. The weight of each mouse and the volume of the tumors generated were recorded every three days. At the end of the experiment, the mice were sacrificed, peripheral blood was collected, and the tumors were excised. Tumor volume was then calculated using the following formula: Volume = 0.5 × length × width^2^. Red blood cell (RBC) count was performed by the Automatic Animal Blood Cell Analyzer (VH30, Huaren, China).

### 2.3. Cell Culture and Transduction

Two human leukemia cell lines (K562 and THP-1) were purchased from the American Type Culture Collection (ATCC). The cells were cultured in RPMI 1640 medium (Wisent, Nanjing, China) containing 10% fetal calf serum (ExCell Bio, Shanghai, China) and 100 U/mL of penicillin/streptomycin (Wisent, Nanjing, China) at 37 °C in a 5% CO_2_ humidified atmosphere. Lentiviruses for over-expressing circ_0059707 were purchased from Shanghai Jikai Biological Co., Ltd. (Shanghai, China). They included a GFP-lentivirus and a lentivirus transfer vector (GV118). Cell transduction was performed according to the manufacturer’s instructions. Then, stably transduced cells were selected using Puromycin dihydrochloride (Beyotime, Shanghai, China) and cell sorting (BD Company, New York, NY, USA).

### 2.4. Cell Growth Assays

The cells (1 × 10^3^) were distributed equally into 24-well plates. The number of viable cells was counted by trypan blue staining at 0, 24, 48, and 72 h.

### 2.5. Cell Apoptosis Assays

The cells (5 × 10^5^) were seeded into 6-well plates containing complete RPMI 1640 culture medium without fetal bovine serum for 48 h. The rate of apoptosis was then detected with an apoptosis detection kit (Annexin V PE/7-AAD BD Biosciences, Franklin Lakes, NJ, USA) by flow cytometry analysis using a FACSCalibur platform (Becton Dickinson, San Jose, CA, USA).

### 2.6. RNA Isolation, Reverse Transcription, and Real-Time Quantitative PCR (qRT-PCR)

The Lymphocyte Separation Medium (TBD Sciences, Tianjin, China) was used to extract mononuclear cells from BM. Total RNA was isolated from 1 × 10^6^ cells using a miScript kit (Qiagen, Dusseldorf, Germany) according to the manufacturer’s instructions and then reverse-transcribed into cDNA using 1 μg of RNA (Takara, Osaka, Japan). The tumor tissue was homogenized by an ultrasound homogenizer, adding TRIZOL (Invitrogen, CA, USA) and chloroform (Xiangde, Xiamen, China) in turn. The samples were centrifuged, treated with isopropanol (Xiangde, Xiamen, China), washed with ethanol and centrifuged. Total RNA was dissolved in RNase-free water. The concentration of RNA was determined by a spectrophotometer, and then RNA was reverse-transcribed into cDNA using 1 μg of RNA (Takara, Japan). The primers for circ_0059707 were 5′-CGTTTGGTGCTTCTCAGATTTC-3′ (forward) and 5′-CATGCCGCTTACCACCATCTAA-3′ (reverse). As a control, we used primers for ABL, whose sequences were 5′-TCCTCCAGCTGTTATCTGGAAGA-3′ (forward) and 5′-TCCAACGAGCGGCTTCAC-3′ (reverse). To detect the expression of miRNA, we proceeded as described above, except that a miScript Reverse Transcription Kit was used to reverse transcribed the RNA into cDNA (Qiagen, Dusseldorf, Germany). The forward primer for miR-1287-5p was 5′-GCGTGCTGGATCAGTGGTT-3′ (forward), and the reverse primer was a universal primer provided by the manufacturer of miScript kit. The primers used as controls were those for human-U6 whose sequences were 5′-GTGCTCCCTGCTTCGGCAGCACATATAC-3′ (forward) and 5′-AAAAATATGGAACGCTTCACGAATTTG-3′ (reverse).

### 2.7. HE Staining

Formalin-fixed subcutaneous tumor masses were embedded in paraffin blocks, cut, and placed on slides. The paraffin sections were dewaxed with xylene and hydrated using an ethanol gradient. Then, staining was carried out using an HE staining kit (Beyotime, Shanghai, China) in accordance with the manufacturer’s instructions. Tissue structures were observed under a light microscope (Nikon, Tokyo, Japan).

### 2.8. Bioinformatics Analysis 

Public gene expression data and full clinical annotations were searched in the Gene-Expression Omnibus databases GSE51908 and GSE142700. GSE51908 contains 3 controls and 18 AML cases. GSE142700 contains 24 controls and 24 AML cases. One control in GSE142700 reported expression levels of all miRNA that were much higher than those found in other control cases; therefore, it was excluded. The StandardScaler function in the ‘Sklearn’ package was used for data standardization, and the ‘matplotlib’ package was applied to obtain a heatmap of gene expression levels via Python software (version 3.7.6).

### 2.9. Statistical Analysis

SPSS 20.0 and GraphPad Prism 5 software were used for data analysis. The relative levels of circ_0059707 expression were calculated with the 2^−ΔΔCT^ method. Categorical variables were analyzed by the Chi-squared test and/or Fisher’s exact tests. The diagnostic capability of circ_0059707 was analyzed using receiver operating characteristic (ROC) curves and area under the curve (AUC) analysis. Survival was analyzed by the Kaplan–Meier method. Univariate and multivariate data were analyzed by Cox regression. Differences in continuous variables between the two groups were compared by Student’s *t*-tests. Differences were considered statistically significant at *p* < 0.05 (two-tailed).

## 3. Results

### 3.1. Relative Expression Levels of Circ_0059707 and Clinical Characteristics of AML

**Figure 1:**
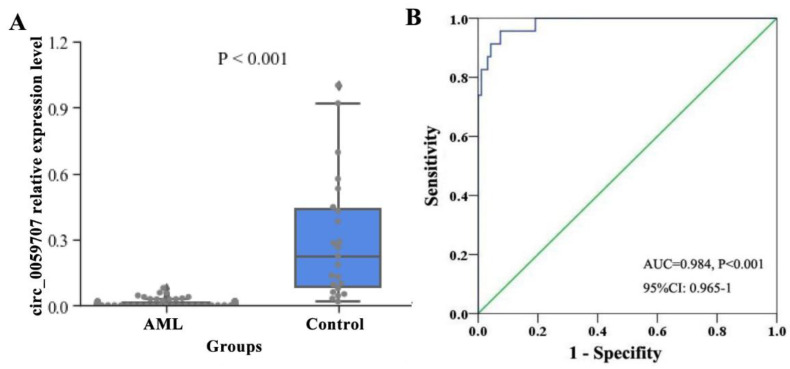
Relative expression levels of circ_0059707 in AML patients. (**A**) Levels of circ_0059707 relative expression in controls and AML patients, as detected by qRT-PCR. (**B**) Discriminative capacity of circ_0059707 expression in AML patients by ROC curve analysis.

The relative expression levels of circ_0059707 in 94 AML patients and 23 controls were detected by qRT-PCR. The median expression levels of circ_0059707 in controls and AML patients were 0.2247 and 0.0057, respectively. The expression levels of circ_0059707 were significantly lower in patients with AML than in controls (*p* < 0.001, Figure 1A). The AUC for circ_0059707 was 0.984 for AML patients (95% confidence interval [CI]: 0.965–1.0, *p* < 0.001) (Figure 1B).

In order to analyze the association between circ_0059707 expression and the clinical characteristics of AML, the patients were divided into a high-circ_0059707 expression group and a low-circ_0059707 expression group, according to a cutoff value of 0.0084 (with a sensitivity of 100% and a specificity of 66%). Comparisons of clinical parameters between the high-expression group and the low-expression group are shown in Table 1. The hemoglobin content (Hb) of peripheral blood in the high-circ_0059707 expression group was significantly higher than that in the-low circ_0059707 expression group (*p* = 0.045). Furthermore, the proportion of bone marrow primordial cells in the high-circ_059707 expression group was significantly lower than that in the low-circ_059707 expression group (*p* = 0.050). 

**Figure 2:**
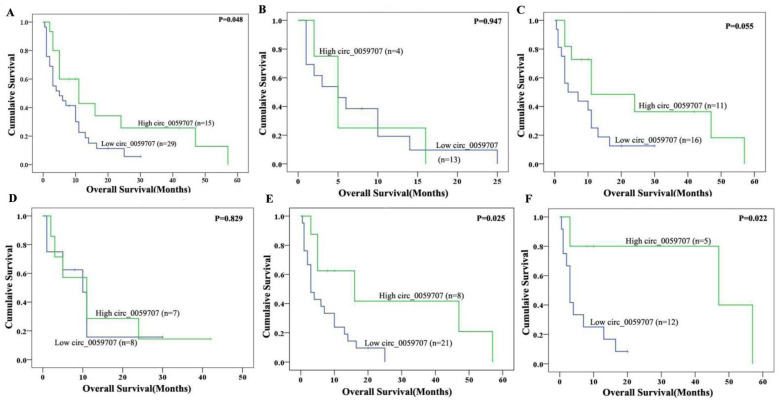
Impact of circ_0059707 expression on overall survival. (**A**) Non-M3 AML patients; (**B**) non-M3 AML patients, age >= 60 years; (**C**) non-M3 AML patients, younger than 60 years; (**D**) non-M3 female AML patients; (**E**) non-M3 male AML patients; (**F**) non-M3 male AML patients, age < 60 years.

A survival analysis of the 58 patients for which survival data were available revealed that there were no significant differences in complete remission (CR) rates when comparing the two groups (*p* = 0.297). However, the overall survival (OS) of patients in the high-circ_0059707 expression group was significantly longer than that of patients in the low-circ_0059707 expression group when considering non-M3 AML patients (*p* = 0.048) (Figure 2A) and AML patients who were younger than 60 years of age; however, these differences were not significant (*p* = 0.055) (Figure 2B,C). The OS of non-M3 male AML patients in the high-circ_0059707 expression group was significantly longer than that of patients in the low-circ_0059707 expression group (*p* = 0.025) (Figure 2D,E). The OS of patients in the high-circ_0059707 expression group was significantly longer than that non-M3 patients who were male and younger than 60 years in the low-circ_0059707 expression group (*p* = 0.022) (Figure 2F). Variables in the univariate analysis with *p* < 0.1 (age, white blood count, risk classification, and circ_0059707 expression) were then included in a multivariate analysis, which subsequently demonstrated that circ_0059707 expression was an independent factor that was related to poor prognosis in non-M3 AML patients (*p* = 0.031) (Table 2). 

### 3.2. Circ_0059707 Inhibited the Growth of Leukemia Cells, Increased Apoptosis, and Inhibited Tumor Growth in Mice

**Figure 3:**
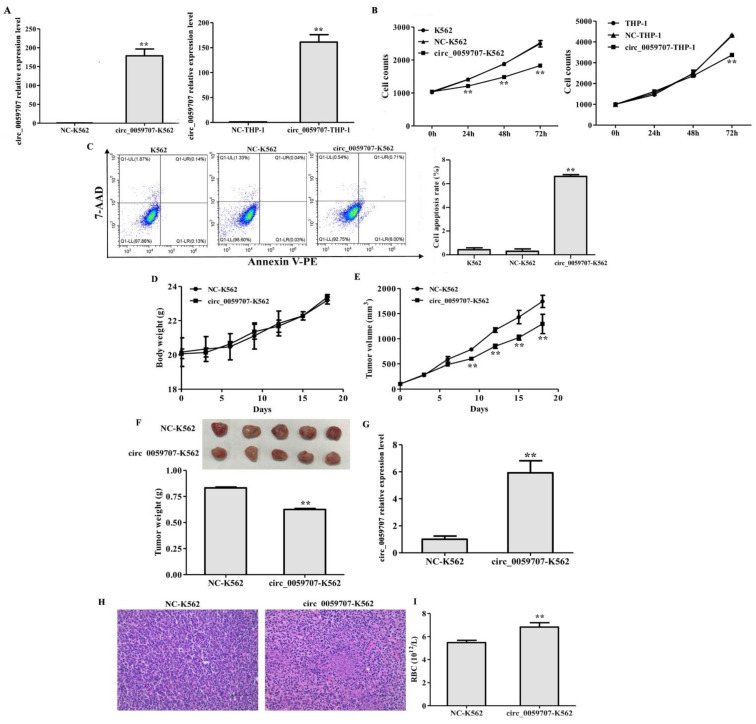
circ_0059707 inhibited the growth of leukemia cells in vitro and in vivo. (**A**) circ_0059707 relative expression, as detected by qRT-PCR. (**B**) The effect of circ_0059707 expression on cell growth. (**C**) The effect of circ_0059707 expression on cellular apoptosis, as detected by flow cytometry. (**D**) Body weight curve for nude mice. (**E**) Tumor volume curve for nude mice. (**F**) Weight of subcutaneous tumors from nude mice. (**G**) Relative expression of levels of circ_0059707 in the tumor tissue, as detected by qRT-PCR. (**H**) HE staining of tumor tissue (200×). (**I**) Red blood cell count in the peripheral blood. **, compared with the NC group, *p* < 0.01.

To investigate the effect of circ_0059707 on leukemia cells, we overexpressed circ_0059707 in K562 and THP-1 cells (Figure 3A). The growth rate of K562 and THP-1 cells was inhibited by circ_0059707 transduction (*p* < 0.01, Figure 3B). Moreover, the rate of apoptosis in cells that had been transduced with circ_0059707 was significantly higher than in normal controls (*p* < 0.01) (Figure 3C). 

To further confirm the role of circ_0059707 in vivo, NC-K562 transduced with a negative control lentivirus and circ_0059707-K562 cells were injected subcutaneously into BALB/c nude mice to allow tumor growth. There was no significant difference in the mean body weight of the animals in the two groups (Figure 3D). The mean tumor volume of mice in the circ_0059707-K562 group was significantly lower than that in the NC-K562 group (*p* < 0.01, Figure 3E). The mean weight of the subcutaneous tumors from the circ_0059707-K562 group was significantly lower than that of tumors from the NC-K562 group (*p* < 0.01, Figure 3F). The levels of circ_0059707 expression in the tumors from each group of mice were detected by qRT-PCR. The analysis showed that the expression levels of circ_0059707 in the circ_0059707-K562 group were significantly higher than those in the NC-K562 group (Figure 3G). 

Tumor tissue harvested from mice in the circ_0059707-K562 group revealed nuclear pyknosis, nuclear fragmentation, nuclear dissolution, and cell necrosis in some areas after HE staining; in addition, we also observed peripheral vascular proliferation and the lack of cell structure in necrotic areas (Figure 3H). Moreover, we observed that the number of peripheral red blood cells in mice of the circ_0059707-K562 group was increased, compared with that of mice inoculated with control cells (Figure 3I). 

### 3.3. Mir-1287-5p Was Up-Regulated by Circ_0059707 

**Figure 4:**
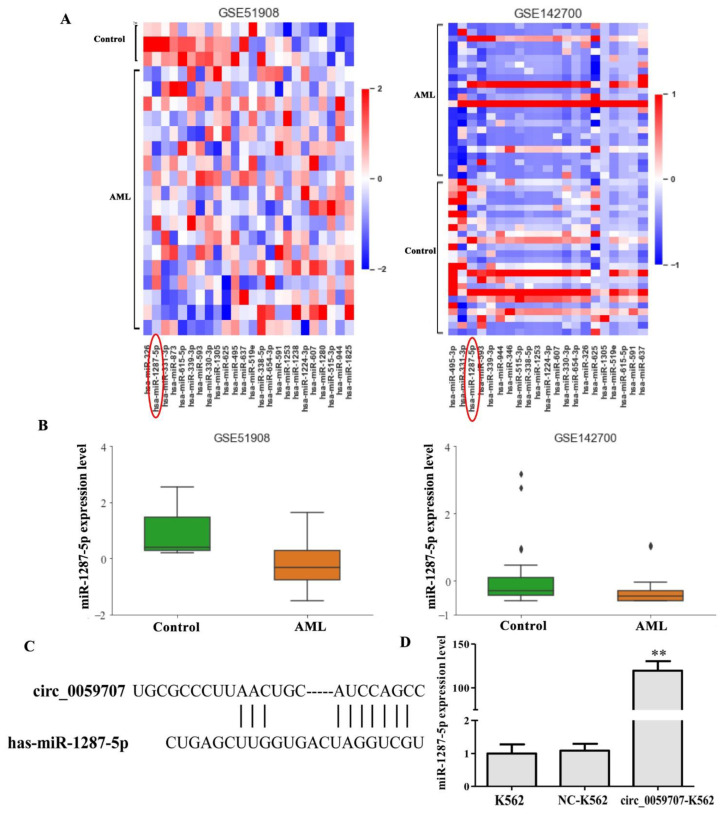
miR-1287-5p may bind to circ_0059707. (**A**) Heatmap of 25 miRNAs in the GSE51908 and GSE142700 datasets from the GEO database. (**B**) The expression levels of miR-1287-5p in controls and AML patients were analyzed in the GSE51908 and GSE142700 datasets from the GEO database. (**C**) Bioinformatics analysis of the binding site between circ_0059707 and miR-1287-5p. (**D**) miR-1287-5p expression levels, as detected by qRT-PCR. **, compared with the NC group, *p* < 0.01.

To determine the possible mechanism involved in the functionality of circ_0059707, we performed additional analyses. First, we analyzed the miRNAs that may bind to circ_0059707 by use of the circInteractome database (https://circinteractome.nia.nih.gov/index.html, accessed on 17 September 2022); this analysis predicted 25 miRNAs that may bind to circ_0059707, including miR-1287-5p, miR-330, miR-495. Then, the expression levels of these miRNAs in AML patients were analyzed using the GEO database (Datasets: GSE51908 and GSE142700). The analysis showed that the levels of miR-1287-5p were lower in AML patients than in controls; this was observed in both the GSE51908 and the GSE142700 datasets (Figure 4A,B). The binding site between circ_0059707 and miR-1287-5p is shown in Figure 4C. Finally, the expression levels of miR-1287-5p were found to be up-regulated in K562 cells that overexpressed circ_0059707 (Figure 4D).

## 4. Discussion

Our research found that circ_0059707, derived from ID1, was down-regulated in AML. A ROC curve analysis indicated the good discriminative capacity of circ_0059707 expression in AML patients. The Hb content in the peripheral blood was significantly higher and the proportion of bone marrow primordial cells was lower in the high-circ_0059707 expression group of patients than in patients in the low-circ_0059707 expression group. Collectively, these findings indicated that high expression levels of circ_059707 may represent a predictor for a good prognosis. There were no significant differences in CR rates between the high- and the low-circ_0059707 expression groups. However, the CR rates was higher in the low-circ_0059707 expression group (44%, 27/62) than that in the high-circ_0059707 expression group (25%, 8/32). This effect could be seen using more samples. Furthermore, the survival analysis revealed that the OS of patients in the high-circ_0059707 expression group was significantly longer than that of patients in the low-circ_0059707 expression group in non-M3 AML patients. A multivariate analysis showed that circ_0059707 was an independent factor associated with a poor prognosis in non-M3 AML patients. These results suggest that circ_0059707 can be used as a new potential target for the diagnosis and prognosis evaluation of non-M3 AML patients. 

CircRNA has been found to play an important role in gene transcription, translation, and epigenetic modifications [26,27,28] and participates in regulating the occurrence of malignancy, tumor proliferation, invasion, and metastasis [29,30,31,32]. CircRNAs have a closed ring structure, without a 5’-end cap or a 3’-polyadenylation tail; therefore, they are not easily recognized and degraded by RNase [33]. In addition, circRNAs exist and function in a highly stable manner in organisms; consequently, circRNAs have good clinical prospects as potential tumor targets [34].

Researchers have already begun to investigate the potential role of circRNA in the pathogenesis of AML. Over recent years, several studies have found that circRNA is closely related to the development of AML. Some circRNAs have been found to be highly expressed in AML patients and act as oncogenes. For example, circ-VIM was found to be significantly up-regulated in AML; moreover, the overexpression of circ-VIM is an independent prognostic factor for OS and leukemia-free survival in AML patients [35]. Other research studies showed that circANAPC7 was significantly up-regulated in AML and participated in the pathogenesis of AML by acting as a sponge for the miR-181 family [36]. The expression of circ-PAN3 was also found to be up-regulated in patients with AML with both refractory and recurrent conditions; the down-regulation of circ-PAN3 restored the sensitivity of THP-1 cells to Adriamycin [37]. The silencing of circ-TASP1 was found to inhibit proliferation and induce apoptosis in AML cells by regulating the miR-515-5p/HMGA2 axis [38]. In another study, circ-PLXNB2, derived from PLXNB2, was shown to be a valuable predictor of the prognosis of patients with AML36. However, some circRNAs are down-regulated in AML and act as tumor suppressors. For example, Hsa_circ_0001947 was found to act as a tumor inhibitor to suppress the proliferation of AML cells via the hsa-miR-329-5p/CREBRF axis [39]. 

In our research, we also found that circ_0059707 inhibited cell growth and promoted apoptosis in leukemia cells. In addition, circ_0059707 inhibited tumor growth in BALB/c nude mice. We found that the tumor volume of mice in the circ_0059707 group was significantly lower than in the control group. Moreover, an increase in the number of red blood cells in the peripheral blood was also detected in the circ_0059707-K562 group, thus suggesting that circ_0059707 may exert positive effects in leukemia patients.

Circ_0059707 came into our sight in the process of studying the miR-29b-ID1 signal pathway. Initially, it was assumed that circ_0059707 might be involved in the DAC drug resistance process via the miR-29b-ID1 pathway and by binding to miR-29b. However, research showed that the overexpression of circ_0059707 led to no significant difference in terms of DAC resistance in K562 and THP-1 cells. In our previous study, we found that miR-29b acts as a tumor suppressor in leukemia cells, while ID1 acts as an oncogene (unpublished data). A previous report found that circ_0059707 has a specific binding site for miR-29b. However, the expression levels of miR-29b in circ_0059707-K562 cells were slightly down-regulated, while the expression levels of ID1 were slightly up-regulated; these findings cannot explain the mechanism by which circ_0059707 inhibited the growth of leukemia cells in vitro and vivo; it is evident that other molecules must also be involved in this process. 

To further investigate the molecular mechanisms responsible for how circ_0059707 can inhibit the growth of leukemia cells, we analyzed 25 miRNAs with common binding sites for circ_0059707 using the circInteractome database. The expression levels of the 25 miRNAs in AML were also analyzed using the datasets GSE51908 and GSE142700 from the GEO database. We found that miR-1287-5p was down-regulated in these two datasets. 

The expression levels of miR-1287-5p in cells overexpressing circ_0059707 were analyzed by qRT-PCR. Compared with the controls, the expression levels of miR-1287-5p were 100-fold higher in cells that overexpressed circ_0059707. We speculate that circ_0059707 inhibits the growth of leukemia cells by promoting the expression of miR-1287-5p. This molecule has been commonly reported to act as a tumor-inhibiting miRNA. Other research has shown that the levels of miR-1287-5p were significantly down-regulated in human breast cancer tissue and that low expression levels were associated with a poor prognosis [40]. miR-1287-5p was also shown to suppress cell proliferation and migration in colorectal cancer [41]. In another study, miR-1287-5p expression was down-regulated in human osteosarcoma and promoted ferroptosis in osteosarcoma cells by inhibiting GPX4 [42].

Taken together, our results indicate that the down-regulation of circ_0059707 is a frequent event and predicts a poor prognosis in de novo AML patients. Furthermore, the overexpression of circ_0059707 can inhibit cell growth, increase cell apoptosis, and inhibit the growth of tumors in mice. Finally, circ_0059707 inhibited the growth of leukemia cells by promoting the expression of miR-1287-5p. These results demonstrate that circ_0059707 may be used as a potential therapeutic and diagnosis target in de novo AML.

## 5. Patents

Inventor: Jichun Ma, Jiang Lin, Jun Qian, Xiangmei Wen, Yu Gu, Ye Jin. A circular RNA hsa_circ_0059707 and its specific amplification primer and application, ZL202110249549.4, authorized on 11 February 2022, invention patent, China.

## Figures and Tables

**Table 1:** Correlation of circ_0059707 expression with clinical characteristics in AML patients.

Patient’s Parameters	Low (n = 62)	High (n = 32)	*p* Value
Sex, male/female	37/25	19/13	0.847
Age, median (range), years ^∆^	54 (15–81)	59 (27–84)	0.127
WBC, median (range), ×10^9^/L ^∆^	49.6 (0.8–528)	55.8 (0.8–207.9)	0.388
Hemoglobin, median (range), g/L ^∆^	84.7 (42–138)	93.3 (58–141)	0.045
Platelets, median (range), ×10^9^/L ^∆^	46.6 (7–192)	66.8 (5–382)	0.103
BM blasts, median (range), % ^∆^	46.1 (0–95.0)	35.8 (0–80.0)	0.050
CR (+/−)	27/22	8/13	0.297
FAB classification			0.433
M0	1	0	
M1	2	0	
M2	24	17	
M3	10	2	
M4	14	5	
M5	6	4	
No data	4	4	
Risk classification			0.273
Low	8 (13%)	3 (9%)	
Intermediate	37 (60%)	23 (72%)	
High	16 (26%)	4 (13%)	
No data	1 (2%)	2 (6%)	
Karyotypes			0.801
normal	29 (47%)	17 (53%)	
t(8;21)	5 (8%)	2 (6%)	
t(16;16)	1 (2%)	0 (0%)	
t(15;17)	10 (16%)	2 (6%)	
t(9;22)	1 (2%)	0 (0%)	
+8	3 (5%)	2 (6%)	
−7/7q-	1 (2%)	0 (0%)	
complex	6 (10%)	3 (9%)	
others	5 (8%)	4 (13%)	
No data	1(2%)	2 (6%)	
Gene mutations			
CEBPA (+/−)	3/49	2/23	0.361
NPM1 (+/−)	10/42	7/20	0.250
FLT3-ITD (+/−)	6/46	2/23	0.322
c-KIT (+/−)	2/50	1/24	0.494
N/K-RAS (+/−)	6/37	2/15	0.417
IDH1/2 (+/−)	2/41	1/16	0.431
DNMT3A (+/−)	4/48	2/23	0.486
U2AF1 (+/−)	0/43	0/17	-
SRSF2 (+/−)	0/43	1/16	0.060
SETBP1 (+/−)	1/34	0/16	0.263
TP53 (+/−)	1/12	1/8	0.420
TET2 (+/−)	0/8	1/4	0.134

Note: ^∆^ median (range).

**Table 2:** Univariate and multivariate analyses of prognostic factors for overall survival in non-M3 AML patients.

	Univariate		Multivariate	
	Hazard Ratio (95% CI)	*p* Value	Hazard Ratio (95% CI)	*p* Value
Circ_0059707 expression	0.500 (0.240–1.044)	0.065	0.441 (0.210–0.929)	0.031
Age	1.841 (0.943–3.594)	0.074	1.401 (0.707–2.778)	0.334
WBC	2.008 (1.012–3.985)	0.046	1.611 (0.756–3.433)	0.009
Risk classification	1.579 (0.888–2.809)	0.019	1.993 (1.199–3.311)	0.008

## Data Availability

The data presented in this study are available on request from the corresponding author.
